# Modified U-NET Architecture for Segmentation of Skin Lesion

**DOI:** 10.3390/s22030867

**Published:** 2022-01-24

**Authors:** Vatsala Anand, Sheifali Gupta, Deepika Koundal, Soumya Ranjan Nayak, Paolo Barsocchi, Akash Kumar Bhoi

**Affiliations:** 1 Chitkara University Institute of Engineering and Technology, Chitkara University, Punjab 140401, India; vatsala.anand@chitkara.edu.in (V.A.); sheifali.gupta@chitkara.edu.in (S.G.); 2 Department of Systemics, School of Computer Science, University of Petroleum & Energy Studies, Dehradun 248007, India; dkoundal@ddn.upes.ac.in; 3 Amity School of Engineering and Technology, Amity University Uttar Pradesh, Noida 201301, India; srnayak@amity.edu; 4 Institute of Information Science and Technologies, National Research Council, 56124 Pisa, Italy; 5 KIET Group of Institutions, Delhi-NCR, Ghaziabad 201206, India; 6 AB-Tech eResearch (ABTeR), Sambalpur 768018, India

**Keywords:** image analysis, segmentation, skin disease, U-Net, deep learning, convolutional neural network

## Abstract

Dermoscopy images can be classified more accurately if skin lesions or nodules are segmented. Because of their fuzzy borders, irregular boundaries, inter- and intra-class variances, and so on, nodule segmentation is a difficult task. For the segmentation of skin lesions from dermoscopic pictures, several algorithms have been developed. However, their accuracy lags well behind the industry standard. In this paper, a modified U-Net architecture is proposed by modifying the feature map’s dimension for an accurate and automatic segmentation of dermoscopic images. Apart from this, more kernels to the feature map allowed for a more precise extraction of the nodule. We evaluated the effectiveness of the proposed model by considering several hyper parameters such as epochs, batch size, and the types of optimizers, testing it with augmentation techniques implemented to enhance the amount of photos available in the PH2 dataset. The best performance achieved by the proposed model is with an Adam optimizer using a batch size of 8 and 75 epochs.

## 1. Introduction

All kinds of microorganisms can cause a variety of skin infections, which can range from moderate to serious. An early diagnosis of a skin illness improves the chances of a successful treatment. Dark patches or birthmarks may appear on the skin as a result of skin illness [1]. The modality used for the diagnosis of skin disease is dermoscopy images. Dermoscopy is a process in which a dermatologist observes a position with a special microscope or magnifying lens. Dermatologist uses a device named dermatoscope, that consists of a high magnifying class, so that a clear picture of the nodule can be seen. In medical imaging, a variety of technologies are employed for the diagnosis of skin disease such as Machine Learning (ML), Deep Learning (DL), Transfer Learning (TL), Ensemble Learning (EL), etc. In ML, a machine is made to learn the tasks, whereas in DL, TL, and EL it learns features directly from the data provided. Improvements in deep learning Convolutional Neural Networks (CNN) have shown promising results in recent years, and they have also emerged as a difficult study topic for categorization in medical image processing [2,3].

Automatically segmenting skin lesions is still a difficult task. Some skin lesions with light pigment have a very similar color and visual patterns in the pigment patches and the surrounding skin regions, making skin lesion segmentation difficult. In addition, the original dermoscopic images have a high resolution, which means that processing them on computers takes a lot of time and resources [4]. All of these factors contribute to the color and texture distribution of the lesions and impede successful learning in the skin lesions themselves. Certain pictures also show hairs and col-or-makers, making skin lesion segmentation more difficult. The segmentation of skin lesions is challenging due to these difficulties.

However, recent studies have shown interest on different variations of DL and CNN models to overcome the above-mentioned difficulties that arise in segmentation [5,6,7,8,9]. Indeed, Yuan et al. [10] presented a completely automated technique for skin lesion segmentation based on 19-layer deep convolutional neural networks that are trained end-to-end and do not require previous data knowledge. They have obtained a value of Jaccard distance of 0.963. In 2017, Yuan et al. [11] proposed convolutional and deconvolutional neural networks using ISBI challenge and obtained a value of Jaccard distance of 0.784. Schaefer et al. [12] combined the enhancement stage with two segmentation algorithms. In the first algorithm, they derived the optimal value of threshold, and in the second algorithm they applied lesion edge detection. Various segmentation techniques developed in skin lesions are shown in literature [13,14]. Shankar et al. [15] showed a histogram thresholding approach to determine the segmented threshold values from the tissues. Pustokhina et al. [16] showed the advantages of edge and region-based techniques that include edge operations and models such as region splitting and merging, etc. Raj et al. [17] used supervised models such as Random forest for skin lesion segmentation by training the model. Anand et al. [18] used pre-processing, segmentation, and classification for the diagnosis of disease. Moreover, they showed that in medical images, the presence of fuzziness and ambiguity produces a wide range of outcomes. Garnavi et al. [19] presented a hybrid threshold-based border detection method to determine nodule areas. Ganster et al. [20] proposed an integrated segmentation technique that is based on thresholding and clustering algorithms. Erkol et al. [21] presented a segmentation method based on the Laplacian-of-Gaussian and active contour methods.

Moreover, in some of the literature, authors have classified skin lesions directly without performing any segmentation of the ROI part. For skin disease classification, various features were used according to the ABCDE rule [22,23]. According to the ABCDE rule, different features such as Asymmetry, Border detection, Color, Diameter and Evolving are used. Recently, UNet architecture was proposed for segmenting lesions and their attributes based on the CNN architecture. A 100-image validation set and a 1000-image test set were derived from ISIC 2018 [24]. Masni et al. [25] used a total of 2750 images from the ISIC’s 2016, 2017, and 2018 datasets. They worked only on three classes—NV, MEL, and AKIEC—and on four pre-trained models—Inception-v3, ResNet-50, Inception-ResNet-v2, and-DenseNet201—and achieved an accuracy of 81.29%, 81.57%, 81.34%, and 73.44%, respectively. Dorj et al. [26] concentrated on utilizing CNN to classify skin cancer. For feature extraction, they employed a pre-trained AlexNet model and used a total of 3753 images. The authors used various classification models for the diagnosis in health applications [27,28,29], and the machine learning methods with ensemble are used with EEG signal [30,31,32,33].

Segmented images of skin lesions have been used by a few researchers to increase classification accuracy. However, image classification, segmentation, and object recognition have all benefited from the increased attention given to Deep Learning (DL) models [34]. In this context, Hang Li et al. [35] proposed dense deconvolutional network on ISBI 2016 and 2017 datasets for skin lesion segmentation based on residual learning. The proposed dense deconvolutional networks reused the learned features from the previous layers, which establishes dense links among all feature maps. By addressing the issue of gradient vanishing, the proposed method strengthens the propagation of multi-level features in the entire network and boosts the performance of skin lesion segmentation greatly. On the 2016 dataset, they obtained a 0.870 value of Jaccard coefficient, and on the 2017 dataset they obtained a 0.765 value of Jaccard coefficient. Yu et al. [36] developed a segmentation and deep classification network with two tiers. They developed a fusion network and obtained an accuracy value of 86.54. Khan et al. [37] proposed a model using pre-processing, segmentation and classification and obtained a segmentation accuracy of 96.8% and 92.1% for the ISIC and PH2 datasets, respectively. Moreover, they obtained a classification accuracy of 97% on the ISIC dataset. Long et al. [38] used the concept of fine tuning with the classification networks AlexNet, GoogleNet, and VGGNet. The authors presented a novel architecture that produces accurate and detailed results by combining semantic information from a deep, coarse layer with appearance information from a shallow, fine layer’s segmentations. Chen et al. [39] combined the methods from deep convolutional neural networks and graphical models for addressing semantic image segmentation. The authors combined the final layers with a fully connected Conditional Random Field. They obtained an IOU accuracy value of 71.6% in the test set. Noh et al. [40] combined a deep deconvolution network, and the proposed technique overcomes the constraints of previous methods based on fully convolutional networks, and the segmentation method frequently finds complex structures and handles objects at many sizes. They achieved an accuracy of 72.5% through ensembling with the fully convolutional network. Wang et al. [41] presented non-local U-Nets that are used with flexible global aggregation blocks. These blocks are used for preserving size in upsampling and downsampling layers. Ibethaz et al. [42] proposed a MultiResUNet architecture, by replacing it with the two convolutional layers. A parameter is assigned for the layers that controls the number of filters of the convolutional layers. They used 97 images ranging from 1349 × 1030 to 1344 × 1024 and resized to 256 × 256, and used the ISIC-2018 dataset. Christ et al. [43] presented a method for the automatic segmentation of lesions in CT abdomen images using cascaded fully convolutional neural networks. They used a two-fold cross validation on the images and obtained a dice score of over 94%. Lin et al. [44] presented a generic multi-path refinement network that explicitly exploits all the information available along the down-sampling process to enable a high-resolution prediction using long-range residual connections. They achieved an intersection-over-union score of 83.4 on the challenging PASCAL VOC 2012 dataset. Novikov et al. [45] used a multi-class configuration, and the ground-truth masks were trained and tested using the publicly accessible JSRT database, which contains 247 X-ray images and can be found in the SCR database. They obtained a Jaccard index value of 95.0%.

The performance results after segmentation increase, and the results obtained are satisfying. From the literature, it can be seen that, when the segmented images are used for classification, the classification accuracy increases.

The major contributions of the study are as follows:

- A modified U-Net architecture has been proposed for the segmentation of lesions from skin disease using dermoscopy images.
- The data augmentation technique has been performed to increase the randomness of images for better stability.
- The proposed model is validated with different optimizers, batch sizes, and epochs for better accuracy.
- The proposed model has been analyzed with various performance parameters such as Jaccard Index, Dice Coefficient, Precision, Recall, Accuracy and Loss.

The rest of the paper is structured as follows: materials and methods are given in Section 2, followed by results and discussions in Section 3, and Section 4 shows the conclusion and future scope.

## 2. Materials and Methods

The proposed model exploits the U-Net architecture for lesion segmentation from skin disease dermoscopy images. The proposed model has been evaluated on the PH2 [46] dataset consisting of 200 skin disease dermoscopy images.

### 2.1. Dataset

**Figure 1:**
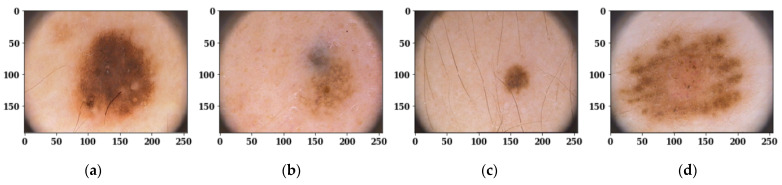
Skin Disease Original Images: (**a**) Image 1; (**b**) Image 2; (**c**) Image 3; (**d**) Image 4.

**Figure 2:**
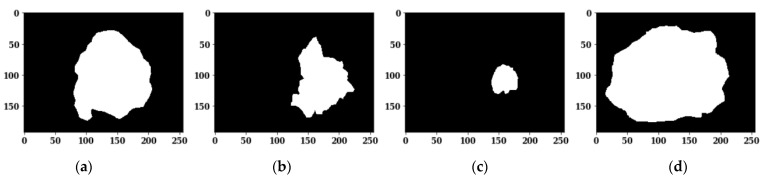
Ground Truth Masks for Respective Original Images: (**a**) Image 1; (**b**) Image 2; (**c**) Image 3; (**d**) Image 4.

The PH2 dataset contains 200 dermoscopy images (160 non-melanomas and 40 melanoma) that are obtained by the Tuebinger Mole Analyzer system using a 20-fold magnification. All images have an approximate size of 192 × 256 pixels. Figure 1 shows the skin diseases’ original images, and Figure 2 shows the ground truth masks for the respective original images.

### 2.2. Data Augmentation

**Figure 3:**
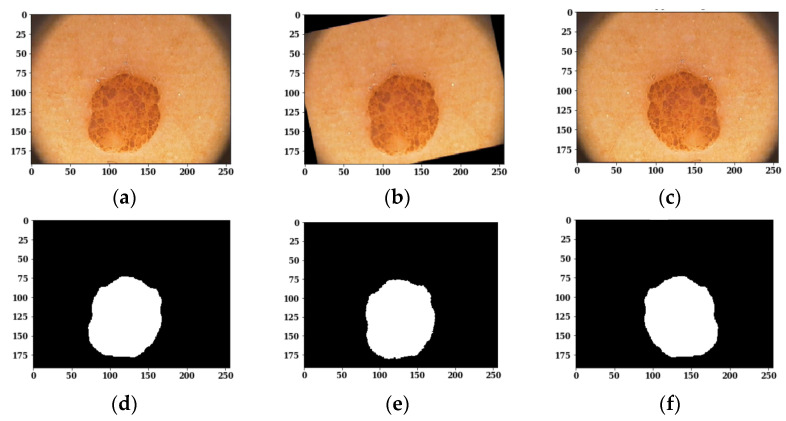
Images and Respective Masks after Data Augmentation Techniques: (**a**) Original Image; (**b**) Rotated Image; (**c**) Flipped Image; (**d**) Original Mask; (**e**) Rotated Mask; (**f**) Flipped Mask.

As the available training dermoscopy images in the dataset are few, offline data augmentation techniques have been implemented to increase the number of sample images. The data augmentation [47] on images is done using different techniques such as flipping and rotation, as shown in Figure 3. The corresponding masks of the augmented images are also shown in Figure 3.

### 2.3. Modified U-Net Architecture

**Figure 4:**
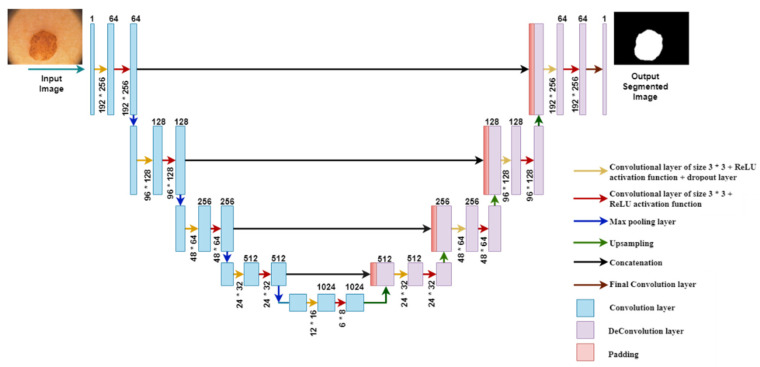
Modified U-Net Architecture.

An enhanced version of the Convolutional Neural Network (CNN) was developed for dealing with biomedical images in which the purpose is not only to categorize whether or not an infection exists but also to identify the infected area [48]. The U-Net architecture consists of two paths. The first one is the contraction path, that is also known as encoder, and the second one is the symmetric expanding path, also known as decoder. Encoder is used to capture the image context, whereas decoder uses transposed convolutions to enable precise localization. In this paper, the proposed modified U-Net architecture has been presented, as shown in Figure 4.

The proposed architecture localizes and distinguishes borders by classifying every pixel; therefore, input and output share the same size. In the encoder part, the convolution layer and the max-pooling layer are applied. In the decoder part, the transposed convolution layer and the simple convolution layer are applied.

During the simulation phase, the Input image undergoes a multilevel decomposition in the encoder path, and the feature maps are reduced with the help of a max pooling layer, which can be seen in Figure 4 as arrows with different colors. The yellow arrows show the convolutional layer of size 3 × 3, ReLU (Rectified Linear Unit) activation function and dropout layer; the red arrows show the convolutional layer of size 3 × 3 and ReLU activation function; the blue arrows show the max-pooling layer; the green arrows show the upsampling with 2 × 2 size; the black arrows show the concatenation of images from contracting and expanding paths; and, finally, the brown arrows show the final convolutional layer with size 1 × 1.

In the contraction path, each and every process consists of two convolutional layers. In the first part, the channel changes from 1 to 64. The blue arrow pointing down shows the max pooling layer that halves down the image from 192 × 256 to 96 × 128. This process is repeated three times and reaches below. Below are the two convolutional layers, but these layers are without max pooling layers. The image has been resized to 12 × 16 × 1024.

In the expanding path, the image is going to be upsized to its original image size. The upsampling technique expands the size of the images, and it is known as transposed convolution. The image is upsized from 12 × 16 to 24 × 32. After that, the image is concatenated with the image from the contracting path. The reason for this is to combine the information from the last layers to get a more accurate prediction. The proposed modified U-Net architecture includes a feature map rectangular in size starting from 192 × 256 in the first layer and 96 × 128 in the second layer. It is downsized again to 48 × 64 in the third layer. Then, it is downsized to 24 × 32 in the fourth layer, and, finally, it is downsized to 6 × 8 in the last layer. Afterwards, the feature map size increases in the expansion path with 24 × 32 in the first layer from the bottom. It is upsized to 48 × 64 in the second layer and to 96 × 128 in the third layer. Finally, the feature map size changes to 192 × 256 in the topmost layer.

After the contraction and expanding process, the architecture reaches the upper level, reshaping the image; the last layer is a convolution layer.

Table 1 shows the parameters of the proposed model, that consists of different convolution layers, input and output image size, filter size, number of filters, and activation function. The total number of parameters for the proposed model are 33,393,669, whereas the total number of trainable parameters are 33,377,795, and non-trainable parameters are 15,874.

## 3. Results and Discussion

This section includes all the results attained by using a modified U-Net model. The model is evaluated on the PH2 dataset. An experimental analysis has been done, from which training accuracy and loss curves are obtained. A detailed description of the performed visual analysis of segmented images and the analysis of confusion matrix parameters is given below.

### 3.1. Result Analysis Based on Different Optimizers

This section includes all the results obtained by using Adam, Adadelta, and SGD optimizers with a batch size of 18 and 100 epochs.

#### 3.1.1. Analysis of Training Loss and Accuracy

**Figure 5:**
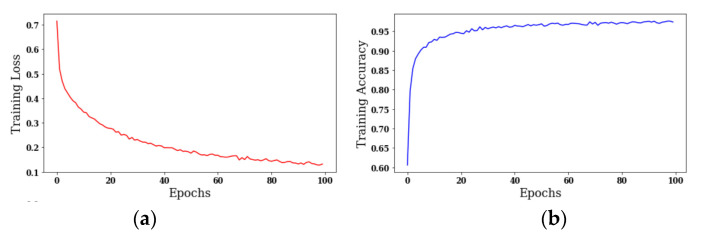

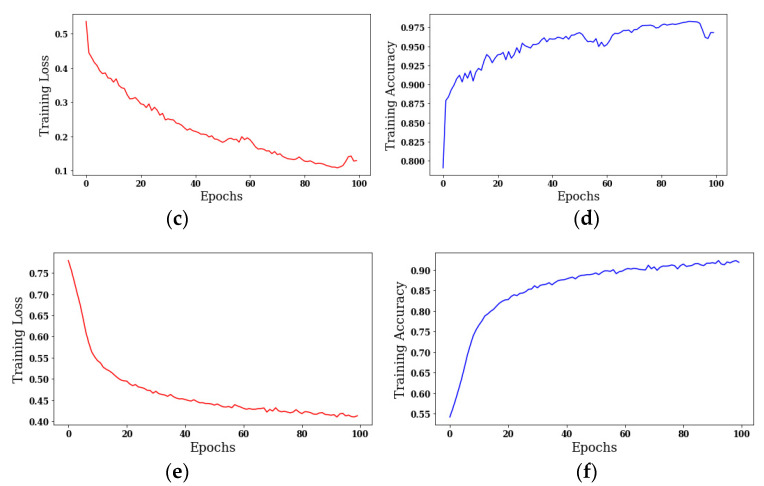
Analysis of training loss and training accuracy: (**a**) Training Loss with SGD optimizer, (**b**) Training Accuracy with SGD optimizer, (**c**) Training Loss with Adam optimizer, (**d**) Training Accuracy with Adam optimizer, (**e**) Training Loss with Adadelta optimizer, (**f**) Training Accuracy with Adadelta optimizer’s Original Image.

The results are taken using different optimizers with a batch size of 18 and 100 epochs. Figure 5 shows the curves of training loss and training accuracy. It is worth noticing that the value of accuracy increases with the number of epochs, and the loss value decreases. The color red shows the training loss, and the color blue shows the training accuracy.

Figure 5a shows the training loss by using the SGD optimizer; the maximum loss value is 0.7, which decreases with the number of epochs. Figure 5b shows the training accuracy in which the maximum accuracy is greater than 0.95 at the 100th epoch. Figure 5c shows the training loss by exploiting the Adam optimizer; the maximum loss value is lower than that of the SGD optimizer. Figure 5d shows the training accuracy in which the maximum accuracy is greater than 0.975 at the 100th epoch. The accuracy for the Adam optimizer outperforms the accuracy at the SGD optimizer. Figure 5e shows the training loss with the Adadelta optimizer, whose maximum value is 0.75, which is higher with respect to the SGD and Adam optimizers. Figure 5f shows the training accuracy, and the value of accuracy is only 0.90. Figure 5 shows that the Adam optimizer outperforms the SGD and Adadelta optimizers in terms of training loss and training accuracy.

#### 3.1.2. Visual Analysis of Segmented Images

**Figure 6:**
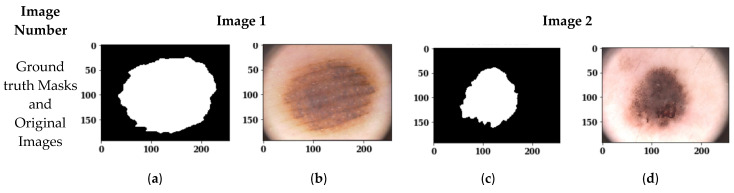

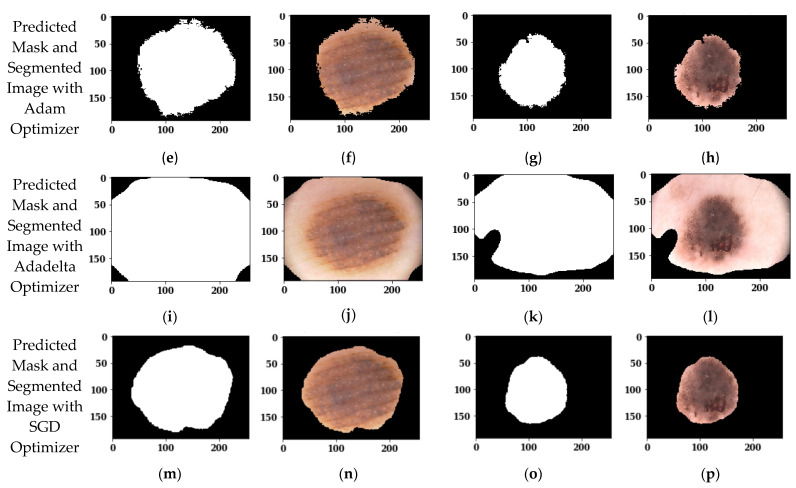
Images segmented with a Batch Size of 18, 100 epochs and different optimizers: (**a**) Ground truth Mask of Original Image 1; (**b**) Original Image 1; (**c**) Ground truth Mask of Original Image 2; (**d**) Original Image 2; (**e**) Predicted Mask of Image 1 with Adam optimizer; (**f**) Segmented Output of Image 1 with Adam optimizer; (**g**) Predicted Mask of Image 2 with Adam optimizer; (**h**) Segmented Output of Image 2 with Adam optimizer and; (**i**) Predicted Mask of Image 1 with Adadelta optimizer; (**j**) Segmented Output of Image 1 with Adadelta optimizer; (**k**) Predicted Mask of Image 2 with Adadelta optimizer; (**l**) Segmented Output of Image 2 with Adadelta optimizer; (**m**) Predicted Mask of Image 1 with SGD optimizer; (**n**) Segmented Output of Image 1 with SGD optimizer; (**o**) Predicted Mask of Image 2 with SGD optimizer; (**p**) Segmented Output of Image 2 with SGD optimizer.

Figure 6 shows the segmented images using the Adam, Adadelta and SGD optimizers with a batch size of 18 and 100 epochs. Figure 6a,c shows the ground truth masks of the original images, and Figure 6b,d shows the original images. Figure 6e,g shows the predicted masks of original images 1 and 2 with the Adam optimizer, whereas Figure 6f,h shows the segmented outputs of original images 1 and 2 with the Adam optimizer. Similarly, Figure 6i–p shows the predicted masks and segmented outputs for the Adadelta and SGD optimizers, respectively. From the visual analysis of these figures, it can be seen that the Adam and SGD optimizers show almost similar results with a batch size of 18 and 100 epochs, whereas the Adadelta optimizer does not follow the profile of the skin lesion; rather, it extracts a complete skin part. So, the Adadelta optimizer cannot be recommended for skin lesion segmentation. To select the best performing optimizer between Adam and SGD, an analysis of these two optimizers is done in Section 3.1.3 using confusion matrix parameters.

#### 3.1.3. Analysis of Confusion Matrix Parameters

In Section 3.1.2, a visual analysis of segmented images is shown, proving that the Adam and SGD optimizers do not have the best results. Now, to see the best performing optimizer, confusion matrix parameters are analyzed. Table 2 shows the comparison of the Jaccard Index, Dice Coefficient, Precision, Recall, Accuracy, and Loss for the modified U-Net model architecture by using the Adam, Adadelta, and SGD optimizers.

**Figure 7:**
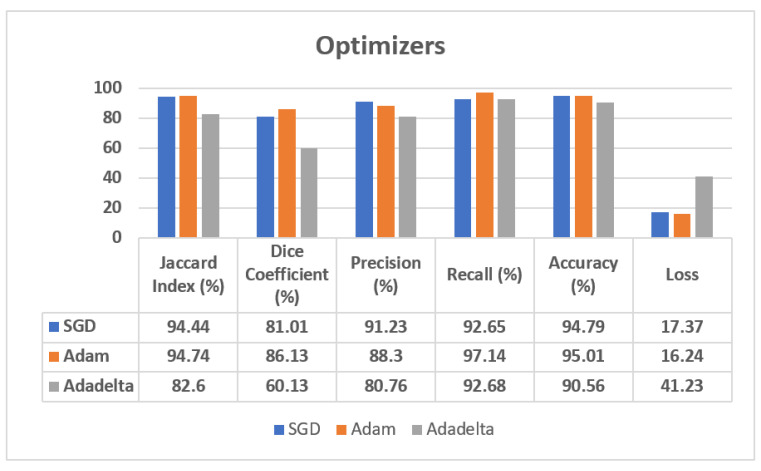
Analysis of confusion matrix parameters on different optimizers.

The validation dataset results, also shown in Figure 7, show that the SGD optimizer reaches the best performance in terms of Precision, with a value of 91.23%, although the Adam optimizer outperforms the SGD optimizer with a 94.74% value of Jaccard Index, 86.13% value of Dice Coefficient, 97.14% value of Recall, 95.01% of accuracy, and 16.24 of loss value. In the case of the Adadelta optimizer, the obtained results show that it is the worst one. Therefore, from these results we can affirm that the Adam optimizer has shown the best results on validation dataset, as it has outperformed on almost all parameters with respect to the SGD and Adadelta optimizers.

Figure 7 shows the analysis of the confusion matrix parameters on the Adam, Adadelta, and SGD optimizers using a validation dataset. From this figure, it can be seen that the Adam optimizer is performing best on almost all the parameters, such as Jaccard Index, Dice Coefficient, Precision, Recall, Accuracy, and Loss. The value of loss is much lower in the case of the Adam optimizer in comparison to the SGD and Adadelta optimizers.

### 3.2. Result Analysis Based on Different Optimizers

From Section 3.1, it is seen that the Adam optimizer has outperformed in comparison to the SGD and Adadelta optimizers with a batch size of 18. Therefore, in this section, the results are calculated using the Adam optimizer on different batch sizes. However, it is possible that the Adadelta and SGD optimizers may provide better results on different combinations of batch size and epochs. In future, these two optimizers can be analyzed for different batch size and epoch combinations. Here, the values of batch sizes used for analyzing the Adam optimizer are 8, 18, and 32 on 100 epochs.

#### 3.2.1. Analysis of Training Loss and Accuracy

**Figure 8:**
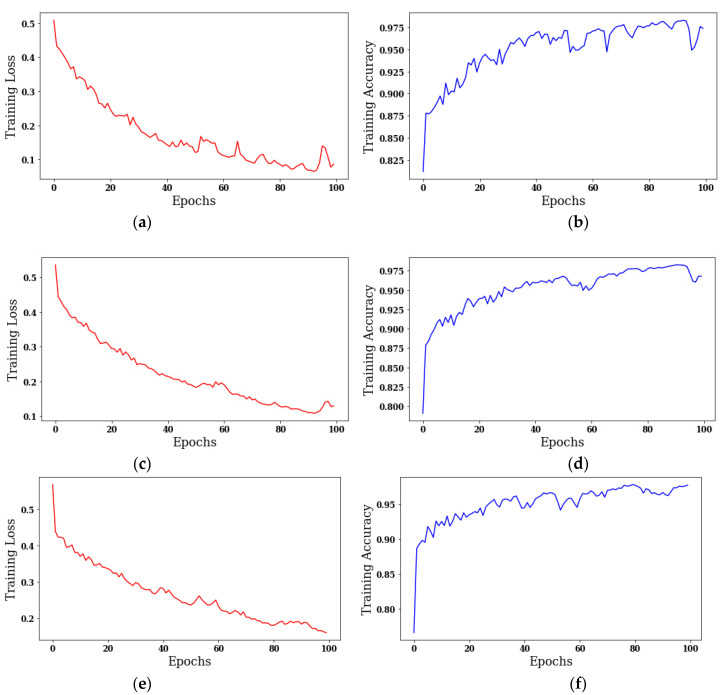
Analysis of training loss and training accuracy: (**a**) Training Loss on batch size 8; (**b**) Training Accuracy on batch size 8; (**c**) Training Loss on batch size 18; (**d**) Training Accuracy on batch size 18; (**e**) Training Loss on batch size 32; (**f**) Training Accuracy on batch size 32.

The results are taken using different batch sizes with the Adam optimizer on 100 epochs. Figure 8 shows the curves of training loss and training accuracy, and from the curves it can be concluded that the value of accuracy increases with the number of epochs, and the loss value decreases. The color red shows the training loss, and the color blue shows the training accuracy.

Figure 8a,c,e shows the training loss on batch sizes 8, 18, and 32, and the loss value is 0.5. Figure 8b,d shows the training accuracy on batch sizes 8 and 18, and the value of accuracy is approximately greater than 0.975. Figure 8f shows the training accuracy, and the value of accuracy is only 0.95 with a batch size of 32.

#### 3.2.2. Analysis of Training Loss and Accuracy

**Figure 9:**
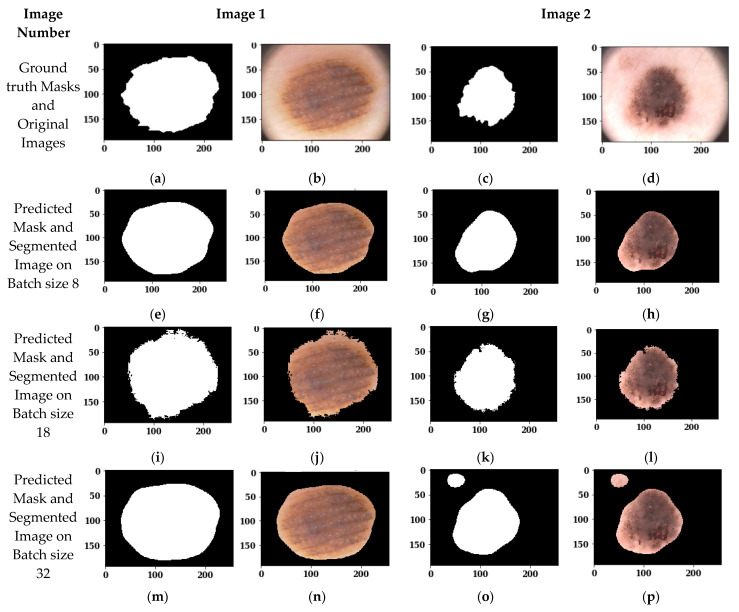
Images segmented on the Adam Optimizer, 100 epochs and different batch sizes: (**a**) Ground truth Mask of Original Image 1; (**b**) Original Image 1; (**c**) Ground truth Mask of Original Image 2; (**d**) Original Image 2; (**e**) Predicted Mask of Image 1 on batch size 8; (**f**) Segmented Output of Image 1 on batch size; (**g**) Predicted Mask of Image 2 on batch size 8; (**h**) Segmented Output of Image 2 on batch size 8; (**i**) Predicted Mask of Image 1 on batch size 18; (**j**) Segmented Output of Image 1 on batch size 18; (**k**) Predicted Mask of Image 2 on batch size 18; (**l**) Segmented Output of Image 2 on batch size 18; (**m**) Predicted Mask of Image 1 on batch size 32; (**n**) Segmented Output of Image 1 on batch size 32; (**o**) Predicted Mask of Image 2 on batch size 32; (**p**) Segmented Output of Image 2 on batch size 32.

Figure 9 shows the segmented images using the Adam optimizer with 100 epochs and a batch size of 8, 18, and 32. Figure 9a,c shows the ground truth masks of original images 1 and 2, and Figure 9b,d shows the original images. Figure 9e,g shows the predicted masks of original images 1 and 2 on batch size 8, whereas Figure 9f,h shows the segmented outputs of original images 1 and 2 on batch size 8. Similarly, Figure 9i–p shows the predicted masks and segmented outputs on batch sizes 18 and 32, respectively. From the visual analysis of the figures, it can be seen that batch sizes 8 and 18 show almost similar results with the Adam optimizer and 100 epochs, whereas batch size 32 does not perform well, since it is not extracting only the lesion part but also the outer part. Therefore, batch size 32 cannot be recommended for skin lesion segmentation. To see the best performing batch size between 8 and 18, the analysis of these two batch sizes, the confusion matrix parameters are evaluated in Section 3.2.3.

#### 3.2.3. Analysis of Confusion Matrix Parameters

In Section 3.2.2, a visual analysis of segmented images is done on different batch sizes, from which batch size 8 and 18 have shown the best results. Now, to see the best performing batch size, the confusion matrix parameters are analyzed. Table 3 shows the analysis of the U-Net model architecture using batch sizes 8, 18, and 32.

**Figure 10:**
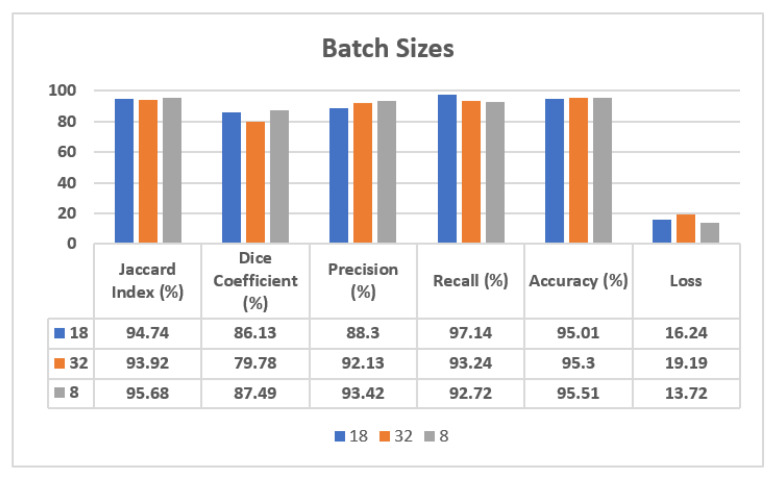
Analysis of confusion matrix parameters on different batch sizes.

In the case of the validation dataset, as also shown in Figure 10, the batch size of 18 has performed best on Recall with a value of 97.14%, although batch size 8 has outperformed and shown a 95.68% value of Jaccard Index, 87.49% value of Dice Coefficient, 93.42% value of Precision, 95.51% value of Accuracy, and a lower loss value, of 13.72. In the case of batch size 32, as already observed with the visual analysis, the performance is lower with respect to the other batch sizes, showing a loss of 19.19. Therefore, from these results it can be seen that batch size 8 has shown the best results on the validation dataset.

Figure 10 shows the analysis of confusion matrix parameters on batch sizes 8, 18, and 32. From the figure it can be seen that batch size 8 is performing best on almost all the parameters, such as Jaccard Index, Dice Coefficient, Precision, Recall, Accuracy, and Loss. The value of loss is much lower in the case of batch size 8 in comparison to batch sizes 18 and 32.

### 3.3. Result Analysis Based on Different Epochs with the Adam Optimizer and Batch Size 8

From Section 3.2, it was seen that batch size 8 has outperformed in comparison to batch sizes 18 and 32 for the Adam optimizer. Therefore, in this section, the results are calculated using batch size 8 with different epochs. However, it is possible that batch sizes 18 and 32 may provide better results on different combinations of epochs. In future, these two batch sizes can be further analyzed with different epochs. Here, the value of epochs used for analyzing the Adam optimizer with batch size 8 are 25, 50, 75, and 100.

#### 3.3.1. Analysis of Confusion Matrix Parameters

**Figure 11:**
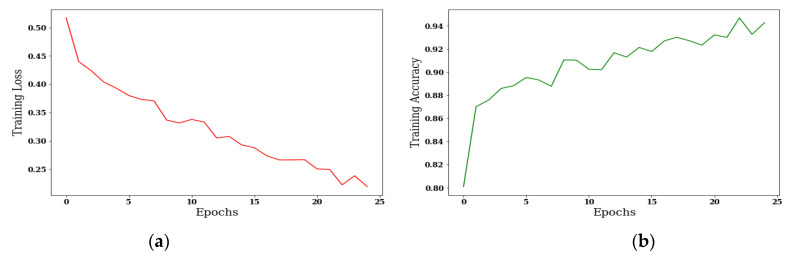

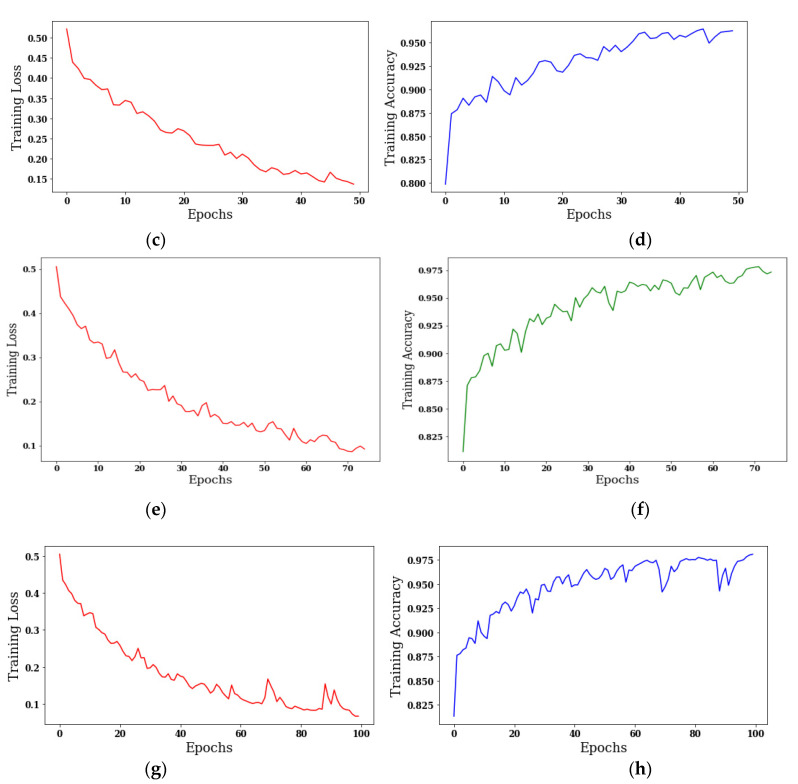
Analysis of training loss and training accuracy: (**a**) Training Loss on 25 epochs; (**b**) Training Accuracy on 25 epochs; (**c**) Training Loss on 50 epochs; (**d**) Training Accuracy on 50 epochs; (**e**) Training Loss on 75 epochs; (**f**) Training Accuracy on 75 epochs; (**g**) Training Loss on 100 epochs; (**h**) Training Accuracy on 100 epochs.

The results are taken using the Adam optimizer on batch size 8 with 25, 50, 75, and 100 epochs. Figure 11 shows the curves of training loss and training accuracy, and from the curves it is concluded that the value of accuracy increases with the number of epochs, and the loss value is decreases.

Figure 11a,c,e,g shows the training loss with 25, 50, 75, and 100 epochs, and the loss value is 0.5; Figure 11b shows the training accuracy on 25 epochs, and the value of accuracy is approximately greater than 0.94. Figure 11f,h shows the training accuracy, and the value of accuracy is only 0.975 on 75 and 100 epochs.

#### 3.3.2. Visual Analysis of Segmented Images

**Figure 12:**
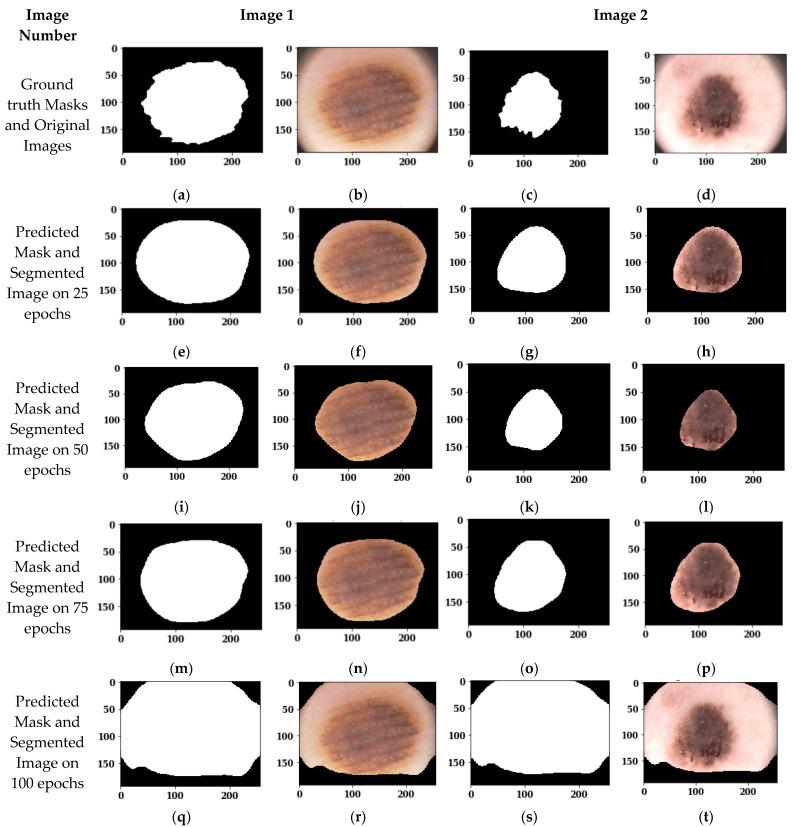
Images segmented on the Adam Optimizer, batch size 8 and different epochs: (**a**) Ground truth Mask of Original Image 1; (**b**) Original Image 1; (**c**) Ground truth Mask of Original Image 2; (**d**) Original Image 2; (**e**) Predicted Mask of Image 1 on 25 epochs; (**f**) Segmented Output of Image 1 on 25 epochs; (**g**) Predicted Mask of Image 2 on 25 epochs; (**h**) Segmented Output of Image 2 on 25 epochs; (**i**) Predicted Mask of Image 1 on 50 epochs; (**j**) Segmented Output of Image 1 on 50 epochs; (**k**) Predicted Mask of Image 2 on 50 epochs; (**l**) Segmented Output of Image 2 on 50 epochs; (**m**) Predicted Mask of Image 1 on 75 epochs; (**n**) Segmented Output of Image 1 on 75 epochs; (**o**) Predicted Mask of Image 2 on 75 epochs; (**p**) Segmented Output of Image 2 on 75 epochs; (**q**) Predicted Mask of Image 1 on 100 epochs; (**r**) Segmented Output of Image 1 on 100 epochs; (**s**) Predicted Mask of Image 2 on 100 epochs; (**t**) Segmented Output of Image 2 on 100 epochs.

Figure 12 shows the segmented images using the Adam optimizer and batch size 8 on different epochs. Figure 12a,c shows the ground truth masks of the original images 1 and 2, and Figure 12b,d shows the original images. Figure 12e,g shows the predicted masks of original images 1 and 2 on 25 epochs, whereas Figure 12f,h shows the segmented outputs of original images 1 and 2 on 25 epochs. Similarly, Figure 12i–t shows the predicted masks and segmented outputs on 50, 75, and 100 epochs, respectively.

From the visual analysis of these figures, it can be seen that 25, 50, and 75 epochs show almost similar results on the Adam optimizer and batch size 8, whereas 100 epochs do not show good results. To see the best performing epochs between 25, 50, and 75, an analysis of these two epochs is done in Section 3.3.3 using confusion matrix parameters.

#### 3.3.3. Analysis of Confusion Matrix Parameters

In Section 3.3.2, a visual analysis of segmented images is done on different epoch values, of 25, 50, 75, and 100. Now, to see the best performing epochs, the confusion matrix parameters are analyzed. Table 4 shows the analysis of the U-Net model architecture using 25, 50, 75, and 100 epochs size and a batch size of 8 with the Adam optimizer.

From Table 4, in the case of the validation dataset, it can be seen that on 25 epochs the value of loss is 28.17, which is very high, followed by a loss value of 23.37 on 50 epochs, whereas on 75 epochs the value of loss becomes lower, i.e., 11.56. Moreover, the values of the Jaccard Index, Dice Coefficient, and Accuracy are increased. Therefore, it can be seen that during the training of the model, there was underfitting on 25 and 50 epochs, due to which the performance parameters values are not good. But at the epoch value of 75, the model is properly trained, so the parameters’ values are also improved. If the model is further trained up to 100 epochs, then the loss value is increased to 165.86. Hence, it can be identified that the proposed model performs best on 75 epochs.

**Figure 13:**
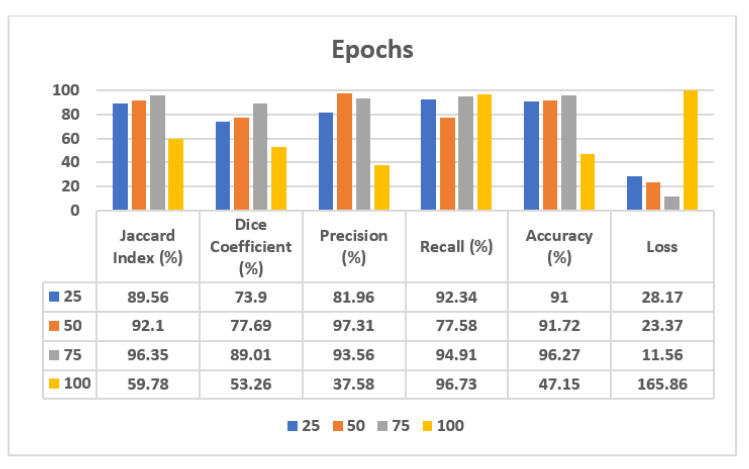
Analysis of confusion matrix parameters on different epochs.

Figure 13 shows the analysis of confusion matrix parameters on 25, 50, 75, and 100 epochs. The results are obtained on the Jaccard Index, Dice Coefficient, Precision, Recall, Accuracy, and Loss. The best value of accuracy is obtained on 75 epochs with a much lower loss.

### 3.4. Comparison with State-of-the-Art Techniques

A comparison of the suggested scheme with other current state-of-the-art methods using dermoscopy images has been performed in terms of both the Jaccard Coefficient and accuracy. Table 5 provides a breakdown of both class-level predictions. This result analysis shows that the proposed framework achieves a superior overall accuracy compared to the state-of-the-art approaches. Jaccard coefficient and accuracy differed from one study to the next, since they employed different datasets (ISBI-2016, ISBI-2017, and PH2). According to Yuan et al. [10,11], the Jaccard Coefficient is 0.963 for the ISBI-2016 dataset and 0.78 for the ISBI-2017 dataset when employing convolutional neural networks.

## 4. Conclusions and Future Scope

Since medical image analysis is one of the challenging tasks which requires various computational techniques in the hierarchy of imaging applications, different types of analysis techniques, including image pre-processing, classification, segmentation, compression, and security, must be taken into account. In the literature, various authors have worked on the segmentation of skin lesions. This study proposed the modified U-Net model architecture for the segmentation of skin lesion in dermoscopy image so that an accurate classification of skin disease can be performed. The dermoscopy images are taken from the PH2 dataset with 200 images. The proposed model has been analyzed with different batch sizes, of 8, 18, and 32, using the Adam, Adadelta, and SGD optimizers and 25, 50, 75, and 100 epochs. The proposed model works best with a batch size of 8, the Adam optimizer, and 75 epochs, having an accuracy of 96.27% and a Jaccard Index of 96.35%. Its Dice Coefficient is coming out as 89.01%. Hence, there is still scope for improving the Dice Coefficient and the Precision of the modified U-Net architecture model. Moreover, in future, segmented images can be used for classification purposes to improve classification accuracy.

## Figures and Tables

**Table 1:** Parameters of the Proposed Model.

S. No.	Layers	Input Image Size	Filter Size	No. of Filter	Activation Function	Output Image Size	Parameters
1	Input Image	192 × 256 × 3	-	-	-	-	-
2	Conv_1	192 × 256 × 3	3 × 3	64	ReLU	192 × 256 × 64	1792
3	Batch Normalization	192 × 256 × 64	-	-	-	-	256
4	Conv 2	192 × 256 × 3	3 × 3	64	ReLU	192 × 256 × 64	36,928
5	Batch Normalization	192 × 256 × 64	-	-	-	-	256
6	MaxPooling	192 × 256 × 64	3 × 3	-	-	96 × 128 × 64	0
7	Conv_3	96 × 128 × 128	3 × 3	128	ReLU	96 × 128 × 128	73,856
8	Batch Normalization	96 × 128 × 128	-	-	-	-	512
9	Conv 4	96 × 128 × 128	3 × 3	128	ReLU	96 × 128 × 128	147,584
10	Batch Normalization	96 × 128 × 128	-	-	-	-	512
11	MaxPooling	96 × 128 × 128	3 × 3	-	-	48 × 64 × 128	0
12	Conv 5	48 × 64 × 256	3 × 3	256	ReLU	48 × 64 × 256	295,168
13	Batch Normalization	48 × 64 × 256	-	-	-	-	1024
14	Conv 6	48 × 64 × 256	3 × 3	256	ReLU	96 × 128 × 128	590,080
15	Batch Normalization	48 × 64 × 256	-	-	-	-	1024
16	MaxPooling	48 × 64 × 256	3 × 3	-	-	48 × 64 × 128	
17	Conv 7	48 × 64 × 256	3 × 3	256	ReLU	96 × 128 × 128	590,080
18	Batch Normalization	48 × 64 × 256	-	-	-	-	1024
19	MaxPooling	48 × 64 × 256	3 × 3	-	-	24 × 32 × 256	0
20	Conv 8	24 × 32 × 512	3 × 3	512	ReLU	24 × 32 × 512	1,180,160
21	Batch Normalization	24 × 32 × 512	-	-	-	-	2048
22	Conv 9	24 × 32 × 512	3 × 3	512	ReLU	24 × 32 × 512	2,359,808
23	Batch Normalization	24 × 32 × 512	-	-	-	-	2048
24	Conv 10	24 × 32 × 512	3 × 3	512	ReLU	24 × 32 × 512	2,359,808
25	Batch Normalization	24 × 32 × 512	-	-	-	-	2048
26	MaxPooling	24 × 32 × 512	3 × 3	-	-	12 × 16 × 512	0
27	Conv 11	12 × 16 × 512	3 × 3	512	ReLU	12 × 16 × 512	2,359,808
28	Batch Normalization	12 × 16 × 512	-	-	-	-	2048
29	Conv 12	12 × 16 × 512	3 × 3	512	ReLU	12 × 16 × 512	2,359,808
30	Batch Normalization	12 × 16 × 512	-	-	-	-	2048
31	Conv 13	12 × 16 × 512	3 × 3	512	ReLU	12 × 16 × 512	2,359,808
32	Batch Normalization	12 × 16 × 512	-	-	-	-	2048
33	MaxPooling	12 × 16 × 512	3 × 3	-	-	6 × 8 × 512	0
34	Upsampling	12 × 16 × 1024	-	-	-	12 × 16 × 1024	0
35	De-Conv 1	12 × 16 × 512	3 × 3	512	ReLU	12 × 16 × 512	4,719,104
36	Batch Normalization	12 × 16 × 512	-	-	-	-	2048
37	De-Conv 2	12 × 16 × 512	3 × 3	512	ReLU	12 × 16 × 512	2,359,808
38	Batch Normalization	12 × 16 × 512	-	-	-	-	2048
39	De-Conv 3	12 × 16 × 512	3 × 3	512	ReLU	12 × 16 × 512	2,359,808
40	Batch Normalization	12 × 16 × 512	-	-	-	-	2048
41	Upsampling	24 × 32 × 512	-	-	-	24 × 32 × 512	0
42	De-Conv 4	24 × 32 × 512	3 × 3	512	ReLU	24 × 32 × 512	2,359,808
43	Batch Normalization	24 × 32 × 512	-	-	-	-	2048
44	De-Conv 5	24 × 32 × 512	3 × 3	512	ReLU	24 × 32 × 512	2,359,808
45	Batch Normalization	24 × 32 × 512	-	-	-	-	2048
46	De-Conv 6	24 × 32 × 256	3 × 3	512	ReLU	24 × 32 × 512	1,179,904
47	Batch Normalization	24 × 32 × 256	-	-	-	-	1024
48	Upsampling	48 × 64 × 256	-	-	-	48 × 64 × 256	0
49	De-Conv 7	48 × 64 × 256	3 × 3	512	ReLU	48 × 64 × 256	590,080
50	Batch Normalization	48 × 64 × 256	-	-	-	-	1024
51	De-Conv 8	48 × 64 × 256	3 × 3	512	ReLU	48 × 64 × 256	590,080
52	Batch Normalization	48 × 64 × 256	-	-	-	-	1024
53	De-Conv 9	48 × 64 × 128	3 × 3	512	ReLU	48 × 64 × 256	295,040
54	Batch Normalization	48 × 64 × 128	-	-	-	-	512
55	Upsampling	96 × 128 × 128	-	-	-	96 × 128 × 128	0
56	De-Conv 10	96 × 128 × 128	3 × 3	512	ReLU	96 × 128 × 128	147,584
57	Batch Normalization	96 × 128 × 128	-	-	-	-	512
58	De-Conv 11	96 × 128 × 64	3 × 3	512	ReLU	96 × 128 × 64	73,792
59	Batch Normalization	96 × 128 × 64	-	-	-	-	256
60	Upsampling	192 × 256 × 64	-	-	-	192 × 256 × 64	0
61	De-Conv 12	192 × 256 × 64	3 × 3	512	ReLU	192 × 256 × 64	36,928
62	Batch Normalization	192 × 256 × 64	-	-	-	-	256
63	De-Conv 13	192 × 256 × 1	3 × 3	512	ReLU	192 × 256 × 1	577
64	Batch Normalization	192 × 256 × 1	-	-	-	-	4
Total Parameters = 33,393,669							
Trainable Parameters = 33,377,795							
Non-Trainable Parameters = 15,874							

**Table 2:** Analysis of Different Optimizers with a batch size of 18 and 100 epochs.

Training Dataset						
Optimizer	Jaccard Index (%)	Dice Coefficient (%)	Precision (%)	Recall (%)	Accuracy (%)	Loss
SGD	96.81	84.60	96.09	96.86	97.77	12.03
Adam	96.42	88.32	92.15	98.50	96.88	11.31
Adadelta	83.90	61.62	86.43	95.82	93.91	38.33
**Testing Dataset**						
	**Jaccard Index (%)**	**Dice Coefficient (%)**	**Precision (%)**	**Recall (%)**	**Accuracy (%)**	**Loss**
SGD	93.98	80.26	90.60	91.64	94.55	17.91
Adam	93.83	84.86	85.89	96.93	94.04	19.19
Adadelta	82.41	59.12	81.08	90.82	90.55	41.54
**Validation Dataset**						
	**Jaccard Index (%)**	**Dice Coefficient (%)**	**Precision (%)**	**Recall (%)**	**Accuracy (%)**	**Loss**
SGD	94.44	81.01	91.23	92.65	94.79	17.37
Adam	94.74	86.13	88.30	97.14	95.01	16.24
Adadelta	82.60	60.13	80.76	92.68	90.56	41.23

**Table 3:** Analysis of Different Batch sizes using the Adam Optimizer.

Training Dataset						
Batch Size	Jaccard Index (%)	Dice Coefficient (%)	Precision (%)	Recall (%)	Accuracy (%)	Loss
8	97.66	90.37	97.10	95.78	97.82	7.90
18	96.42	88.32	92.15	98.50	96.88	11.31
32	94.79	80.87	92.93	96.08	96.45	17.02
**Testing Dataset**						
	**Jaccard Index (%)**	**Dice Coefficient (%)**	**Precision (%)**	**Recall (%)**	**Accuracy (%)**	**Loss**
8	95.72	87.29	92.04	94.12	95.77	12.54
18	93.83	84.86	85.89	96.93	94.04	19.19
32	92.92	78.37	89.19	93.23	94.34	21.41
**Validation Dataset**						
	**Jaccard Index (%)**	**Dice Coefficient (%)**	**Precision (%)**	**Recall (%)**	**Accuracy (%)**	**Loss**
8	95.68	87.49	93.42	92.72	95.51	13.72
18	94.74	86.13	88.30	97.14	95.01	16.24
32	93.92	79.78	92.13	93.24	95.30	19.19

**Table 4:** Analysis of different epochs using the Adam optimizer and batch size 8.

Training Dataset						
Epochs	Jaccard Index (%)	Dice Coefficient (%)	Precision (%)	Recall (%)	Accuracy (%)	Loss
25	88.69	73.72	81.72	93.69	91.58	27.71
50	93.51	79.81	98.74	81.03	93.62	18.99
75	97.66	90.79	95.95	96.89	97.79	7.79
100	59.97	53.07	37.62	96.75	47.37	164.86
**Testing Dataset**						
	**Jaccard Index (%)**	**Dice Coefficient (%)**	**Precision (%)**	**Recall (%)**	**Accuracy (%)**	**Loss**
25	89.72	72.95	80.05	94.58	91.64	27.60
50	93.10	78.97	96.55	81.10	93.35	19.44
75	95.57	87.41	90.62	95.23	95.47	13.78
100	57.38	50.65	35.46	96.86	43.25	181.64
**Validation Dataset**						
	**Jaccard Index (%)**	**Dice Coefficient (%)**	**Precision (%)**	**Recall (%)**	**Accuracy (%)**	**Loss**
25	89.56	73.90	81.96	92.34	91.00	28.17
50	92.10	77.69	97.31	77.58	91.72	23.37
75	96.35	89.01	93.56	94.91	96.27	11.56
100	59.78	53.26	37.58	96.73	47.15	165.86

**Table 5:** Comparison of the Proposed Model with State-of-the-Art Techniques.

Ref	Technique Used	Dataset	Performance Parameters
Yuan et al. [10]	19-layer Deep Convolution Network	ISBI-2016	Jaccard Coefficient = 0.963
		PH2	
Yuan et al. [11]	Convolutional-Deconvolutional neural Network	ISBI-2017	Jaccard Coefficient = 0.784
Hang Li et al. [28]	Dense Deconvolutional Network	ISBI-2016	Jaccard Coefficient = 0.870
		ISBI-2017	Jaccard Coefficient = 0.765
Yu et al. [29]	Convolution Network	ISBI-2016	Accuracy = 0.8654
		ISBI-2017	
Khan et al. [30]	Convolution Network	ISIC	Accuracy = 0.968
		PH2	Accuracy = 0.921
**Proposed Model**	**Modified U-Net**	**PH2**	**Jaccard Coefficient = 0.976**
	**Architecture**		**Accuracy = 0.977**

## Data Availability

Not applicable as the study did not require ethical approval. The data (PH2 dataset) is available in a publicly accessible repository.

## References

[1] Anand V., Gupta S., Koundal D. (2022). Skin Disease Diagnosis: Challenges and Opportunities. Advances in Intelligent Systems and Computing, Proceedings of the Second Doctoral Symposium on Computational Intelligence, Lucknow, India, 5 March 2022.

[2] Shinde P.P., Seema S. A Review of Machine Learning and Deep Learning Applications. Proceedings of the 2018 Fourth International Conference on Computing Communication Control and Automation (ICCUBEA).

[3] Goyal A. (2014). Around 19 Crore Indians Likely to Suffer from Skin Diseases by 2015-Notes Frost & Sullivan. https://www.freepressjournal.in/business-wire-india-section/around-19-crore-indians-likely-to-suffer-from-skin-diseases-by-2015-notes-frost-sullivan.

[4] Liu L., Tsui Y.Y., Mandal M. (2021). Skin lesion segmentation using deep learning with auxiliary task. J. Imaging.

[5] Liu L., Mou L., Zhu X.X., Mandal M. (2020). Automatic skin lesion classification based on mid-level feature learning. Comput. Med. Imaging Graph..

[6] Li Y., Shen L. (2018). Skin lesion analysis towards melanoma detection using deep learning network. Sensors.

[7] Singh V.K., Abdel-Nasser M., Rashwan H.A., Akram F., Pandey N., Lalande A., Presles B., Romani S., Puig D. (2019). FCA-Net: Adversarial learning for skin lesion segmentation based on multi-scale features and factorized channel attention. IEEE Access.

[8] Yang X., Zeng Z., Yeo S.Y., Tan C., Tey H.L., Su Y. (2017). A novel multi-task deep learning model for skin lesion segmentation and Classification. arXiv.

[9] Xie Y., Zhang J., Xia Y., Shen C. (2020). A mutual bootstrapping model for automated skin lesion segmentation and classification. IEEE Trans. Med. Imaging.

[10] Yuan Y., Chao M., Lo Y.-C. (2017). Automatic skin lesion segmentation using deep fully convolution networks with Jaccard distance. IEEE Trans. Med. Imaging.

[11] Yuan Y. (2017). Automatic skin lesion segmentation with fully convolutional-deconvolutional networks. arXiv.

[12] Schaefer G., Rajab M.I., Celebi M.E., Iyatomi H. (2011). Colour and contrast enhancement for improved skin lesion segmentation. Comput. Med. Imaging Graph..

[13] Bi L., Kim J., Ahn E., Kumar A., Fulham M., Feng D. (2017). Dermoscopic image segmentation via multi-stage fully convolutional networks. IEEE Trans. Biomed. Eng..

[14] Liu X., Song L., Liu S., Zhang Y. (2021). A review of deep-learning-based medical image segmentation methods. Sustainability.

[15] Shankar K., Zhang Y., Liu Y., Wu L., Chen C.H. (2020). Hyperparameter tuning deep learning for diabetic retinopathy fundus image classification. IEEE Access.

[16] Pustokhina I.V., Pustokhin D.A., Gupta D., Khanna A., Shankar K., Nguyen G.N. (2020). An effective training scheme for deep neural network in edge computing enabled Internet of Medical Things (IoMT) systems. IEEE Access.

[17] Raj R.J.S., Shobana S.J., Pustokhina I.V., Pustokhin D.A., Gupta D., Shankar K. (2020). Optimal feature selection-based medical image classification using deep learning model in internet of medical things. IEEE Access.

[18] Anand V., Koundal D. (2019). Computer-assisted diagnosis of thyroid cancer using medical images: A survey. Proceedings of ICRIC 2019.

[19] Garnavi R., Aldeen M., Celebi M.E., Varigos G., Finch S. (2011). Border detection in dermoscopy images using hybrid thresholding on optimized color channels. Comput. Med. Imaging Graph..

[20] Ganster H., Pinz P., Rohrer R., Wildling E., Binder M., Kittler H. (2001). Automated melanoma recognition. IEEE Trans. Med. Imaging.

[21] Erkol B., Moss R.H., Stanley R.J., Stoecker W.V., Hva-tum E. (2005). Automatic lesion boundary detection in dermoscopy images using gradient vector flow snakes. Skin Res. Technol..

[22] She Z., Liu Y., Damatoa A. (2007). Combination of features from skin pattern and ABCD analysis for lesion classification. Ski. Res. Technol..

[23] Celebi M.E., Wen QU A.N., Iyatomi Shimizu Zhou H., Schaefer G. (2015). A state-of-the-art survey on lesion border detection in dermoscopy images. Dermoscopy Image Anal..

[24] Koohbanani N.A., Jahanifar M., Tajeddin N.Z., Gooya A., Rajpoot N. (2018). Leveraging transfer learning for segmenting lesions and their attributes in dermoscopy images. arXiv.

[25] Masni A., Mohammed A., Kim D.H., Kim T.S. (2020). Multiple skin lesions diagnostics via integrated deep convolutional networks for segmentation and classification. Comput. Methods Programs Biomed..

[26] Dorj U.O., Lee K.K., Choi J.Y., Lee M. (2018). The skin cancer classification using deep convolutional neural network. Multimed. Tools Appl..

[27] Mishra S., Tripathy H.K., Mallick P.K., Bhoi A.K., Barsocchi P. (2020). EAGA-MLP—An enhanced and adaptive hybrid classification model for diabetes diagnosis. Sensors.

[28] Roy S., Poonia R.C., Nayak S.R., Kumar R., Alzahrani K.J., Alnfiai M.M., Al-Wesabi F.N. (2022). Evaluating the Usability of mHealth Applications on Type-2 Diabetes Mellitus using various MCDM Models. Healthcare.

[29] Srinivasu P.N., Bhoi A.K., Nayak S.R., Bhutta M.R., Woźniak M. (2021). Block-chain Technology for Secured Healthcare Data Communication among the Non-Terminal nodes in IoT architecture in 5G Network. Electronics.

[30] Satapathy S.K., Bhoi A.K., Loganathan D., Khandelwal B., Barsocchi P. (2021). Machine learning with ensemble stacking model for automated sleep staging using dual-channel EEG signal. Biomed. Signal Process. Control..

[31] Pramanik M., Pradhan R., Nandy P., Bhoi A.K., Barsocchi P. (2021). Machine Learning Methods with Decision Forests for Parkinson’s Detection. Appl. Sci..

[32] Saxena U., Moulik S., Nayak S.R., Hanne T., Sinha Roy D. (2021). Ensemble-Based Machine Learning for Predicting Sudden Human Fall Using Health Data. Math. Probl. Eng..

[33] Garg M., Gupta S., Nayak S.R., Nayak J., Pelusi D. (2021). Modified Pixel Level Snake using Bottom Hat Transformation for Evolution of Retinal Vasculature Map. Math. Biosci. Eng..

[34] Li H., He X., Zhou F., Yu Z., Ni D., Chen S., Wang T., Lei B. (2018). Dense deconvolutional network for skin lesion segmentation. IEEE J. Biomed. Health Inform..

[35] Kathiresan S., Sait A.R.W., Gupta D., Lakshmanaprabu S.K., Khanna A., Pandey H.M. (2020). Automated detection and classification of fundus diabetic retinopathy images using synergic deep learning model. Pattern. Recogn. Lett..

[36] Yu Z., Jiang X., Zhou F., Qin J., Ni D., Chen S., Lei B., Wang T. (2018). Melanoma recognition in dermoscopy images via aggregated deep convolutional features. IEEE Trans. Biomed. Eng..

[37] Khan A.H., Iskandar D.A., Al-Asad J.F., El-Nakla SAMIRAnd Alhuwaidi S.A. (2021). Statistical Feature Learning through Enhanced Delaunay Clustering and Ensemble Classifiers for Skin Lesion Segmentation and Classification. J. Theor. Appl. Inf. Technol..

[38] Long J., Shelhamer E., Darrell T. Fully convolutional networks for semantic segmentation. Proceedings of the IEEE Conference on Computer Vision and Pattern Recognition 2015.

[39] Chen L.C., Papandreou G., Kokkinos I., Murphy K., Yuille A.L. (2014). Semantic image segmentation with deep convolutional nets and fully connected crfs. arXiv.

[40] Noh H., Hong S., Han B. Learning deconvolution network for semantic segmentation. Proceedings of the IEEE International Conference on Computer Vision.

[41] Wang Z., Zou N., Shen D., Ji S. (2020). Non-local u-nets for biomedical image segmentation. Proc. AAAI Conf. Artif. Intell..

[42] Ibtehaz N., Rahman M.S. (2020). MultiResUNet: Rethinking the U-Net architecture for multimodal biomedical image segmentation. Neural Netw..

[43] Christ P.F., Elshaer M.E.A., Ettlinger F., Tatavarty S., Bickel M., Bilic P., Rempfler M., Armbruster M., Hofmann F., D’Anastasi M. (2016). Automatic liver and lesion segmentation in CT using cascaded fully convolutional neural networks and 3D conditional random fields. Proceedings of the International Conference on Medical Image Computing and Computer-Assisted Intervention.

[44] Lin G., Milan A., Shen C., Reid I. Refinenet: Multi-path refinement networks for high-resolution semantic segmentation. Proceedings of the IEEE Conference on Computer Vision and Pattern Recognition.

[45] Novikov A.A., Lenis D., Major D., Hladůvka J., Wimmer M., Bühler K. (2018). Fully convolutional architectures for multiclass segmentation in chest radiographs. IEEE Trans. Med. Imaging.

[46] Mendonca T., Celebi M., Marques J. (2015). Ph2: A Public Database for the Analysis of Dermoscopic Images. Dermoscopy Image Analysis.

[47] Shorten C.C., Khoshgoftaar M.T. (2019). A Survey on Image Data Augmentation for Deep Learning. J. Big Data.

[48] Ronneberger O., Fischer P., Brox T. (2015). U-net: Convolutional networks for biomedical image segmentation. In International Conference on Medical image computing and computer-assisted intervention. arXiv.

